# The Expression of CD14^+^CD16^+^ Monocyte Subpopulation in Coronary Heart Disease Patients with Blood Stasis Syndrome

**DOI:** 10.1155/2013/416932

**Published:** 2013-06-27

**Authors:** Ye Huang, Jing-Shang Wang, Hui-jun Yin, Ke-ji Chen

**Affiliations:** ^1^Emergency Department, Xiyuan Hospital, China Academy of Chinese Medical Sciences, Beijing 10091, China; ^2^Department of Cardiovascular Disease, Xiyuan Hospital, China Academy of Chinese Medical Sciences, Beijing 100091, China

## Abstract

Blood stasis syndrome (BSS), a comprehensive pathological state, is one of the traditional Chinese medicine syndromes of coronary heart disease (CHD). In our previous study, we investigated that Fc**γ**RIIIA (also called CD14^+^CD16^+^ monocyte subpopulation) is one of the differentially expressed genes related to CHD patients and its possible role in the atherosclerotic formation and plaque rupture. However, whether or not the deregulation of CD14^+^CD16^+^ monocyte subpopulation expression is implicated in the pathogenesis of CHD patients with BSS has not yet been elucidated. In this study, we found that there was no significant difference between CHD patients with BSS and non-BSS in CD14^+^CD16^+^ monocyte subpopulation at gene level. Moreover, the protein level of CD14^+^CD16^+^ monocyte subpopulation in CHD patients with BSS was increased significantly when compared to the CHD patients with non-BSS. Additionally, the level of inflammatory cytokines downstream of CD14^+^CD16^+^ monocyte subpopulation such as TNF-**α** and IL-1 in sera was much higher in CHD patients with BSS than that in CHD patients with non-BSS. Taken together, these results indicated that CD14^+^CD16^+^ monocyte subpopulation was implicated in the pathogenesis of CHD patients with BSS, which may be one of the bases of the essence of BSS investigation.

## 1. Introduction

The way of disease-syndrome combination is an important style to diagnose and treat disease in traditional Chinese medicine (TCM) clinical practice today. Study on the blood stasis syndrome (BSS) is the most active field of integration of traditional and western medicine research in China [1]. To normalize and standardize the BSS, the way of disease-syndrome combination is used to explore the essence of BSS, which will be the inevitable tendency in the future. Study on CHD with BSS initiated by research team of Chen keji is the model of the way of disease-syndrome combination. In our previous study, we found that Fc receptor III A of immunoglobulin G (Fc*γ*RIIIA, also called CD14^+^CD16^+^ monocyte subpopulation) is one of the differentially expressed genes related to coronary heart disease (CHD) patients using the oligonucleotide microarray technique [2], and high level of Fc*γ*RIIIA in CHD patients observed previously was verified by both mRNA level and its protein content [3]. Our recent study suggested an important role of Fc*γ*RIIIA in the atherosclerotic formation by elevating the adhesive efficiency of monocytes to HUVECs *in vitro*, by increasing expression of inflammatory cytokines and also by kindling the atherosclerotic plaque destabilization in ApoE^−/−^ mouse [4]. Additionally, we also investigated that traditional Chinese medicine of activation of blood and dissolving stasis, effective components of Chuanxiong Rhizome and Red Peony Root, could stabilize the atherosclerotic plaque by suppressing inflammation, and its target was relative with Fc*γ*RIIIA [5]. However, whether or not the deregulation of the expression of CD14^+^CD16^+^ monocyte subpopulation is implicated in the pathogenesis of CHD patients with BSS has not yet been elucidated.

## 2. Methods

### 2.1. Patients and Healthy Control

All patients with coronary heart disease were the inpatients of Beijing Anzhen Hospital, from May 2010 to December 2010, diagnosed by a diameter stenosis of at least 50% from standard selective coronary angiography [6, 7]. These CHD patients were selected into blood stasis syndrome (BSS) group and non-BSS group based on the standard diagnostic criteria established by the Special Committee of Promoting Blood Circulation and Removing Blood Stasis, Chinese Association of Integrative Medicine [8]. Patients with severe valvulopathy, serious primary diseases such as liver or kidney dysfunction, malignant tumors, medication history of antiplatelet therapy, and women in pregnancy or lactation stage were excluded from enrollment. Forty age- and sex-matched healthy individuals from the physical examination center of Xiyuan Hospital were selected as a control group. These individuals were without any history of chest pain or evidence of cardiac or other systemic disease verified by history examination, chest film, electrocardiogram, and blood routine examination, and none was taking any medication. Our study was in accordance with the Helsinki Declaration, with ethical approval granted by the Ethics Committee at Xiyuan Hospital, China Academy of Chinese Medical Sciences, and a written informed consent was obtained from all study participants. The clinical characteristics of the participants are shown in Table 1.

### 2.2. Blood Samples

Peripheral blood was collected from the CHD patients with BSS/non-BSS and healthy subjects under standardized conditions. Blood from CHD patients was drawn before coronary angiography was performed. For the analyses of cytometry or mRNA expression or flow cytometry, 2 mL ethylenediaminetetraacetate- (EDTA-) anticoagulated blood was taken and immediately analyzed. For the detection of inflammatory cytokines by ELISA assay, blood was centrifuged at 3,000 rpm for 20 min, and serum was frozen at −80°C until analysis.

### 2.3. RT-PCR

Fc*γ*RIIIA mRNA expression was investigated by the quantitative real-time polymerase chain reaction (PCR) assay. Total RNA samples were extracted from leukocytes using Trizol reagent (Invitrogen, USA) according to the manufacturer's protocol. The purity and integrity of RNA were determined on a UV spectrophotometer (Eppendorf, Germany) by 260–280 nm absorbance ratio and agarose gel electrophoresis (1.5%) and ethidium bromide staining, respectively. cDNA preparations were performed at 42°C for 1 h, with reverse transcriptase, 2 *μ*L RNase, 2 *μ*L of an oligo (dT) primer, and 10 mM of each dNTP in a total volume of 50 *μ*L of 1x first strand cDNA synthesis buffer, incubated at 70°C for 10 min. PCR assays were carried out in a PCR (ABI 7500, USA). 1.5 *μ*L of cDNA mixture was subjected to amplification in a 20 *μ*L mixture. The following primer sequences with the predicted size were used for amplification: Fc*γ*RIIIA: forward, 5′-TGTTCAAGGAGGAAGACCCT-3′, reverse, 5′-GAAGTAGGAGCCGCTGTCTT-3′; GAPDH: forward, 5′-GGGTGTGAACCATGAGAAGT-3′, reverse, 5′-GGCATGGACTGTGGTCATGA-3′. PCR conditions were as follows: initial denaturation at 94°C for 15 min followed by 40 cycles of denaturation for 15 s at 94°C, annealing at 60°C for 34 sec, extending at 72°C for 15 sec, and a final extension at 72°C for 10 min. Glyceraldehydes-3-phosphate dehydrogenase (GAPDH) was used as an internal control in all PCR reactions. The PCR products were subjected to 2% agarose gel electrophoresis. The relative mRNA expression level of the target gene in each individual was calculated using the comparative cycle time (*C*_t_) method [9].

### 2.4. Flow Cytometry

Fc*γ*RIIIA protein level was assessed by flow cytometry. Ethylenediaminetetraacetic-Acid- (EDTA-) anticoagulated peripheral blood (PB) samples were collected from all patients and healthy controls for flow cytometric analysis performed previously described [10]. Briefly, PB samples were stained with saturating concentrations of fluorescein-isothiocyanate- (FITC-) conjugated anti-CD14 monoclonal antibody (mAb) (BD Biosciences, Lot: 74003) and phycoerythrin- (PE-) conjugated anti-CD16 mAb (BD Biosciences, Lot: 73903) or isotype-matched control mAb for 20 min at room temperature in the dark. After erythrocytes were lysed by incubation with lysing solution for 8 min, PB mononuclear cells were resuspended in PBS with 1% fetal calf serum. The surface expression of CD14 and CD16 on PB monocytes was performed by a fluorescence-activated cell sorter (FACS) cytometer (Becton Dickinson). The test data were obtained by FS versus SS gate and analyzed by Expo32 special software. Monocytes were identified by gating CD14^+^ events, and all additional analyses were performed on this population. Fc*γ*RIIIA protein content defined by the percentage of CD16 on the monocyte population (CD14^+^/CD16^+^%) was measured.

### 2.5. Enzyme-Linked Immunosorbent Assay (ELISA)

Concentrations of TNF-*α* (R&D, USA, Lot: 1007143), IL-1 (R&D, USA, Lot: 1007155), and soluble CD14 (sCD14) (R&D, USA, Lot: 1010179) in sera were determined by double-antibody sandwich avidin-biotin peroxidase complex enzyme-linked immunosorbent assay (ABC-ELISA), according to manufacturer's instructions.

### 2.6. Statistical Analysis

All data are expressed as mean ± SD. The SPSS Statistics 15.0 package was utilized to analyze the data. Differences among groups were analyzed using the one-way analysis of variance (ANOVA), followed by multiple comparisons by LSD test. Difference was considered significant at *P* < 0.05.

## 3. Results and Discussion

BSS is a pathological state, which is the outward manifestation of some certain pathological stage of various diseases. Due to lack of objective diagnosis criteria, the essence of BSS is studied into the bottleneck stage. Recently, based on the way of disease-syndrome combination, much effective exploration of the essence of BSS was the foundation of BSS objective diagnosis criteria construction. During the past 50 years, we found a correlation between CHD with BSS and inflammatory, hemodynamics, platelet, and microcirculation [11].

**Figure 1:**
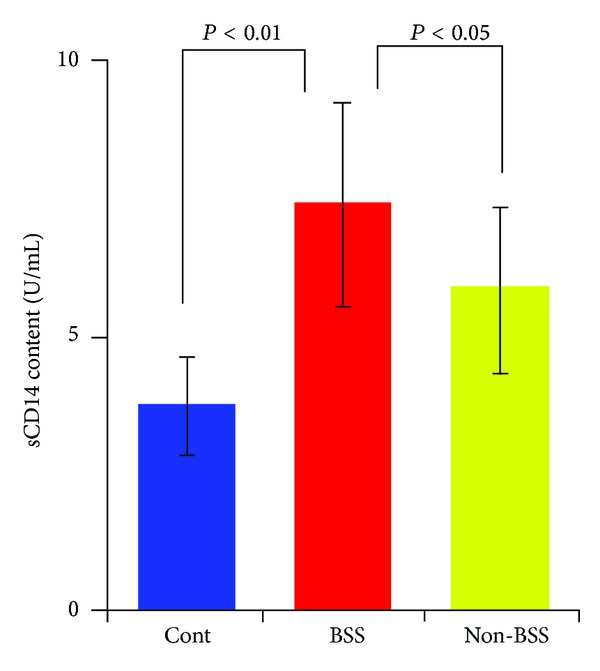
The significant level of soluble CD14 in sera in CHD patients with BSS by ELSIA assay. Results were presented as mean ± SD.

**Figure 2:**
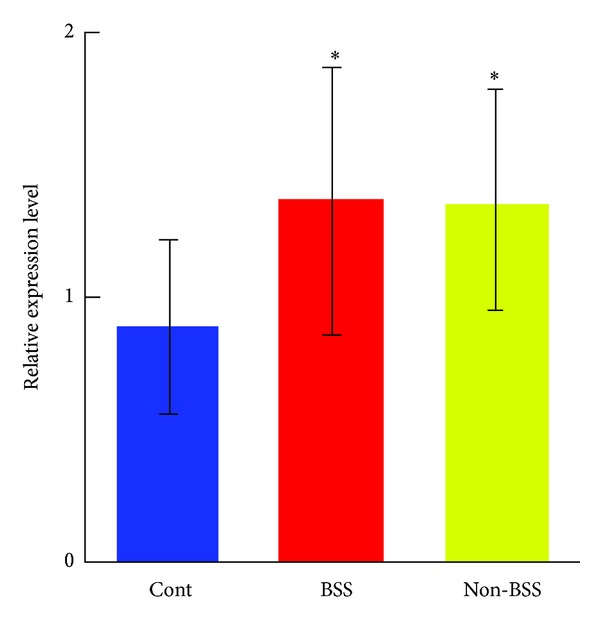
The mRNA level of CD14^+^CD16^+^ monocyte subpopulation in leukocytes in CHD patients with BSS by qRT-PCR. *P < 0.01 compared to the control group. Results were presented as mean ± SD.

**Figure 3:**
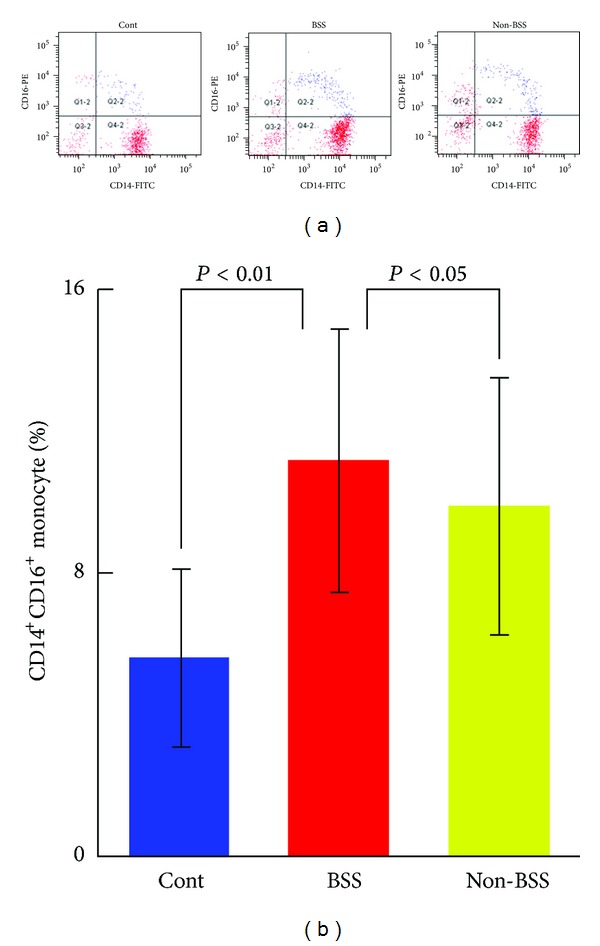
The protein level of CD14^+^CD16^+^ monocyte subpopulation in CHD patients with BSS by FACS analysis. (a) Representation data of FACS analysis of CD14^+^CD16^+^ monocyte subpopulation. Whole peripheral blood samples from patients and healthy individuals were stained with FITC-conjugated anti-CD14 antibody and PE-conjugated anti-CD16 antibody, followed by FACS. (b) The percentage of CD14^+^CD16^+^ monocoyte subpopulation in the whole CD14-positive cells. Results were presented as mean ± SD.

Atherosclerosis, a chronic inflammatory immune state, is chiefly responsible for the development of CHD. Various leukocytes have been shown to influence atherogenesis. Monocytes and their descendant macrophages are central protagonists in the development of atherosclerosis [12]. Monocyte migration to the vessel wall is an initial event in the growth of atherosclerotic lesions [13]. Once monocytes are activated, adhesion to endothelial cells was induced by the transform of phenotype, which led to myocardium injury, inducing the proinflammatory cytokines such as TNF-*α* and IL-1 synthesis to initiate the inflammatory cascade reaction and oxidative stress injury, producing matrix metalloproteinase (MMP) and releasing many media to induce plaque instability and even fracture [14, 15]. Therefore, monocytes played the key role in the chronic inflammation-immunoreaction of the arterial vessels. In our study, we investigated that there was no significant difference of monocyte count based on CBC count among CHD patients with BSS, non-BSS, and healthy control (Table 1). However, the level of sCD14 which is the indicator of activated monocyte [16] was obviously increased in CHD patients with BSS, compared to non-BSS and healthy control (Figure 1). The increased level of sCD14 in sera in CHD patients with BSS indicated monocytes activation. To demonstrate the correlation between deregulated expression of CD14^+^CD16^+^ monocyte subpopulation and pathogenesis of CHD with BSS, its mRNA expression at the leukocyte level was assessed. As shown in Figure 2, relative expression level of CD14^+^CD16^+^ monocyte subpopulation in both CHD patients with BSS and non-BBS was largely increased by 99% and 77%, respectively, compared to the healthy control (*P* < 0.01). However, there was no significant difference of this relative expression level between CHD patients with BSS and non-BSS (Figure 2). The expression of biological traits was controlled by gene, and the biological traits were reflected by protein. To investigate whether or not the expression change of CD14^+^CD16^+^ monocyte subpopulation in both CHD patients with BSS and non-BSS at its protein level, therefore, we further analyzed the protein level of CD14^+^CD16^+^ on monocyte member using 2-color immunofluorescent staining (Figure 3(a)). The FACS results showed that the protein level of CD14^+^CD16^+^ on monocyte member was significantly increased in the CHD patients with BSS, when compared to the CHD patients with non-BSS and the healthy control (*P* < 0.01  or *P* < 0.05, Figure 3(b)).

**Figure 4:**
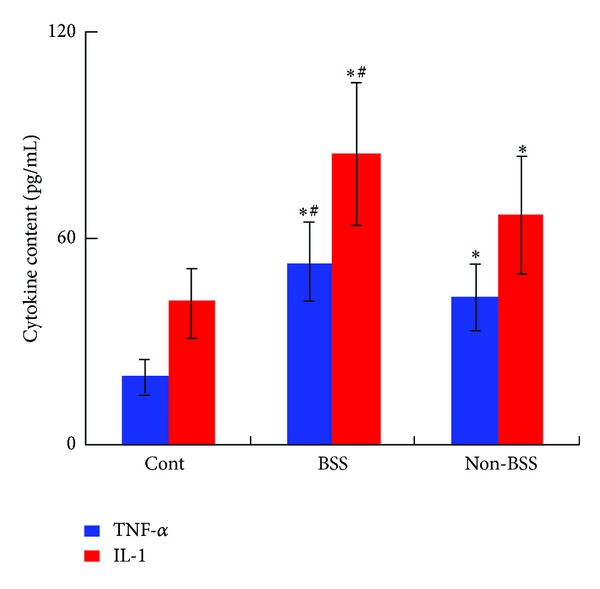
The changes of inflammatory cytokines of TNF-*α* and IL-1 in sera in CHD patients with BSS. *P < 0.01 compared to the control group. ^#^ *P* < 0.05 compared to the CHD patients with non-BSS. Results were presented as mean ± SD.

TNF-*α* is one of the cytokines with various biological activation, and one previous study confirms that increased level of TNF-*α* was existed in the monocyte/macrophage, smooth muscle cells, and endothelial cells in the atherosclerotic plaque, and this high level was correlated to the severity of atherosclerosis [17]. IL-1 was released by active monocyte/macrophage, which induced the releasing of many cytokines and growth factors by monocyte/macrophage and the expression of adhesion molecules such as ICAM-1. Additionally, IL-1 could stimulate vascular endothelium to produce many inflammatory factors such as TNF-*α*, aggravated local inflammatory reaction, and promote the development of atherosclerosis [18, 19]. Additionally, previous studies indicated that the circulation of CD14^+^CD16^+^ monocytes could spontaneously produce TNF and IL-1 [20], which could provoke cell proliferation and migration of smooth muscle cells and macrophages in the atherosclerotic plaque [21]. Therefore, protein level of TNF-*α* and IL-1 in sera was also assessed in our study by ELISA. The significant increased serum of TNF-*α* and IL-1 level was observed in CHD patients with BSS and non-BSS compared to the healthy control (*P* < 0.01, Figure 4), and the level of TNF-*α* and IL-1 in CHD patients with BSS was much higher than that in CHD patients with non-BSS (*P* < 0.05, Figure 4).

In the current study, we investigated that there were monocyte activation and an increased CD14^+^CD16^+^ monocyte subpopulation at protein level and its downstream inflammatory cytokines such as TNF-*α* and IL-1 in sera in CHD patients with BSS. Herein, we presumed that monocyte activation in the CHD patient with BSS induced the phenotype of monocyte member transforming to CD14^+^CD16^+^ monocyte subpopulation which was involved in the pathogenesis of CHD with BSS.

## 4. Conclusion

Overall, the present work confirmed the correlation between increased CD14^+^CD16^+^ monocyte subpopulation at protein level and CHD patients with based on the way of disease-syndrome combination. Thus, the increased CD14^+^CD16^+^ monocyte subpopulation and its downstream inflammatory cytokines in CHD patient with BSS indicated that CD14^+^CD16^+^ monocyte subpopulation was one of the sensitive markers in the pathogenesis of CHD with BSS.

## Figures and Tables

**Table 1:** Characteristics of the CHD patients with BSS/non-BSS and healthy individuals participated in the study.

	CHD patients			
	BSS patients	Non-BSS patients	Healthy control	*P* value
	(*n* = 50)	(*n* = 50)	(*n* = 40)	
Age (year)	53.00 ± 6.43	52.92 ± 6.03	49.50 ± 8.71	0.507
Sex (male/female)	36/14	33/17	28/12	0.804
BMI (kg/m^2^)	25.50 ± 2.77	25.10 ± 2.57	24.29 ± 1.57	0.509
SAP (*n*)	12	13	—	—
ACS (*n*)	38	37	—	—
UAP (*n*)	31	31	—	—
AMI (*n*)	7	6	—	—
Hypercholesterolemia (>230 mg/dL) (yes/no)	7/43	5/45	—	—
Hypertension (yes/no)	31/19	30/20	—	—
Diabetes (yes/no)	20/30	20/30	—	—
Monocyte count (mmol/L)	0.38 ± 0.11	0.37 ± 0.20	0.36 ± 0.11	0.891

BMI: body mass index; SAP: stable angina pectoris; ACS: acute coronary syndrome; UAP: unstable angina; AMI: acute myocardial infarction. Data are expressed as mean ± SD.
